# 肺段切除术中联合多种修补材料对于减少术后肺漏气的回顾性研究

**DOI:** 10.3779/j.issn.1009-3419.2020.101.41

**Published:** 2020-10-20

**Authors:** 望 张, 洪磊 许, 伟 闻, 俊 王, 亮 陈, 全 朱

**Affiliations:** 210029 南京，南京医科大学第一附属医院，江苏省人民医院胸外科 Department of Thoracic Surgery, Jiangsu Provincial People's Hospital, The First Affiliated Hospital of Nanjing Medical University, Nanjing 210029, China

**Keywords:** 微孔多聚糖止血粉, 肺段切除术, 可吸收性聚乙醇酸补片, 纤维蛋白胶, 肺漏气, Microporous polysaccharide hemostatic powder, Pulmonary segmentectomy, Polyglycolic acid, Fibrin glue, Pulmonary air leakage

## Abstract

**背景与目的:**

解剖性肺段切除术在肺结节及早期肺癌中的应用愈加广泛，术后肺漏气是其术后常见并发症之一。本研究旨在探究胸腔镜下精准肺段切除手术术中修补材料的应用对于减少术后肺漏气的效果。

**方法:**

本研究纳入2018年8月1日-2019年7月31日在江苏省人民医院胸外科拟行胸腔镜下肺段切除术的入院患者，根据术中处理段间交界面时使用材料的差异进行分组：采用微孔多聚糖止血粉+纤维蛋白粘合胶+可吸收性聚乙醇酸补片的患者划分为A组，而采用纤维蛋白粘合胶+可吸收性聚乙醇酸补片的患者划分为B组。收集并记录所有患者的术前基础信息及术后的每日胸腔引流量、胸腔引流管的留置时间、拔除胸腔引流管前的胸片、拔除胸腔引流管后的胸片、血常规以及术后住院时间，并分析术中修补材料的应用对术后肺漏气的影响。

**结果:**

两组患者胸腔引流管留置时间（*P*=0.019）、术后住院时间（*P*=0.017）具有统计学差异。

**结论:**

在肺段切除术中段间交界面的处理上使用微孔多聚糖止血粉+纤维蛋白粘合胶+可吸收性聚乙醇酸补片相比于使用纤维蛋白粘合胶+可吸收性聚乙醇酸补片能更好地减少患者术后漏气的发生及缩短术后住院时间。

## Abstract (en)

**Background and objective:**

Anatomical segmentectomy is more and more widely used in lung nodules and early stage lung cancer. Postoperative lung air leakage is one of the common complications after surgery. This study aimed to explore the effect of the application of repair materials in precise segmentectomy under thoracoscopy in reducing postoperative lung air leakage.

**Methods:**

This study included patients admitted to the Department of Thoracic Surgery of Jiangsu Provincial People's Hospital who were scheduled to undergo thoracoscopic segmentectomy from August 1, 2018 to July 31, 2019. According to the difference of the materials used in the treatment of the inter-segment interface during the operation: patients who used microporous polysaccharide hemostatic powder+fibrin adhesive glue+absorbable polyglycolic acid patch were divided into group A, and the patients with fibrin adhesive+absorbable polyglycolic acid patch were divided into group B. The preoperative basic information of all patients and the daily postoperative chest drainage volume, the indwelling time of the chest drainage tube, the chest radiograph before the chest drainage tube is removed, the chest radiograph after the chest drainage tube is removed, blood routine and postoperative hospital stay were collected and recorded, and the effect of the application of intraoperative repair materials on postoperative lung air leakage was analyzed.

**Results:**

There were statistically significant differences in the indwelling time of thoracic drainage tube (*P*=0.019) and postoperative hospital stay (*P*=0.017) between the two groups.

**Conclusion:**

Compared with the use of the fibrin glue+absorbability polyglycolic acid patch, the use of microporous polysaccharide hemostatic powder+fibrin glue+absorbability polyglycolic acid patch in the treatment of the inter-segment interface during segmentectomy can better reduce the incidence of postoperative air leakage and shorten the postoperative hospital stay.

数据^[1, 2]^表明，全球肺癌发生率及死亡率所有恶性肿瘤中居于首位。其中绝大多数为非小细胞肺癌（non-small cell lung cancer, NSCLC）。近年来，随着低剂量螺旋计算机断层扫描（computed tomography, CT）筛查的推广，肺结节及早期NSCLC的发现率呈现上升趋势，早期NSCLC的手术患者不断增加^[3, 4]^。目前，对于早期NSCLC的主要治疗方式依然是手术治疗^[5]^。随着胸腔镜下肺段切除术的不断完善，精准肺段切除术以保存更多的有效肺容积，对肺功能影响较小的优点越来越多的被医生与患者们作为治疗早期NSCLC的重要手段^[6]^。

虽然胸外科医生们在探究如何减少术后肺漏气的道路上不曾中止，然而术后肺漏气却依然是其术后常见并发症之一^[7]^。Cerfolio等^[8]^研究表明，术中使用生物蛋白胶喷洒肺断面，可有效减少术后肺漏气时间及住院时间。Nomori等^[9]^研究表明纤维蛋白粘合胶+三层可吸收性聚乙醇酸（polyglycolic acid, PGA）补片可大大减少术后漏气时间。然而由于解剖性肺段切除术分离靶段时更易造成肺组织的损伤，且段间平面较大，而单片PGA补片较小且价格昂贵，若用纤维蛋白粘合胶+三层PGA补片的修补方式，将大大增加患者的住院费用。因此如何更加有利于减少患者的术中耗材费用及术后肺漏气的发生一直都是临床医生的长久的难题。

微孔多糖止血粉由淀粉多糖和羧基壳聚糖乳化交联共聚而成，是一种具有微孔结构的复合多糖，具有亲水性分子筛的作用，通过浓缩血液中的固形成分到微粒表面，形成凝胶状基质而加速自然止血。作为一种新的植物性快速止血材料，在胸外科手术中可显著缩短止血时间以及减少术后胸管输出和术后输血需求的功效已得到临床实验证明^[10]^。我们最早在临床实践中偶然发现使用微孔多聚糖止血粉+纤维蛋白粘合胶+PGA补片联合使用覆盖创面减少了肺段术后肺漏气的发生，为进一步验证，我们在离体猪肺肺段切除动物模型上进行了研究，结果表明三者的联合使用可以显著提高对抗气道压力的能力，减少肺漏气的发生^[11]^。为了观察其临床效果，我们进行了回顾性研究。

## 1 资料与方法

### 1.1 一般资料

纳入2018年8月1日-2019年7月31日江苏省人民医院胸外科单个医疗组以“肺结节”为主诉且拟行胸腔镜下肺段切除术的入院患者。根据患者手术录像及病历进行筛选。术前排除标准：①暂不适宜手术治疗，如急性感染、不稳定性心绞痛、急性脑梗塞、深静脉血栓形成、多发性肿瘤病变等；②肺部情况复杂，可能影响术中操作及术后相关指标的判断：如严重慢性阻塞性肺疾病、反复发作的气胸、既往肺部手术病史、既往结核病史、既往脓胸/胸膜炎病史、解剖变异如肺隔离症/支气管畸形及肺纤维化等；③多发结节，无法单独采用胸腔镜下单个肺段切除术达到治疗效果；④患者或家属拒绝手术治疗，或其他原因未手术者。术前检查结果的收集：所有患者均进行了常规入院检查，包括血常规、生化、心电图、肺功能、血气分析、胸部CT等相关检查。收集并记录患者术前的基础信息（性别、年龄等）及部分实验室检查指标（肺功能检查及肿瘤标志物）。

### 1.2 手术方法

所有患者的手术方式均为解剖性肺段切除术。患者采用可视喉镜行双腔气管导管插管，由同一组手术医师完成手术。手术均顺利完成，无术中并发症。术前均采用三维支气管血管成像（three-dimensional bronchoangiography, 3D-CTBA）进行肺结构三维重建、术前规划、术中导航。手术切口采用三孔法，第7肋间腋中线1 cm切口置入胸腔镜，主操作孔选择腋前线第4或第5肋间3 cm切口，副操作孔位于第7肋间肩胛下角线与腋后线之间1.5 cm切口。采用丝线结扎细小肺动静脉分支，较大的肺动静脉分支采用腔镜直线切割缝合器切断（强生电动腔镜关节头直线切割吻合器（PSE45, ECR45 M, ECR 45W）。靶段、靶亚段支气管采用腔镜直线切割缝合器切断（强生ECR 45G）。采用“改良膨胀萎陷法” ^[12]^来确定段间交界面，采用锥式肺段切除理念，切割缝合器适形裁剪技术^[12]^分割段间交界面。段间交界面的分离近段门处使用电刀分离（电凝，功率40W），外周使用腔镜直线切割缝合器进行分割（强生ECR 45G、ECR 45G）。使用温无菌蒸馏水在恒定气道压力20 cm H_2_O的情况下测试有无肺漏气。术中肺漏气的标准^[13]^：0级：无漏气；1级：偶尔见小气泡；2级：连续串珠样小气泡；3级：大量成簇状堆积气泡。放置单根24 F胸腔引流管连接胸腔闭式引流。

### 1.3 实验分组

根据术中处理段间交界面的差异分为两组，A组采用微孔多聚糖止血粉+纤维蛋白粘合胶+PGA补片覆盖创面，B组采用纤维蛋白粘合胶+PGA补片覆盖创面。术中排除标准：①发现胸腔广泛黏连，需大规模分离烙断时，该患者不纳入回顾性研究队列，因为可能有额外的漏气风险及更多的术后引流量；②术中存在意外情况，如重要血管或神经损伤、大气道损伤，或出现麻醉意外等不可控因素时；③术中情况复杂，手术中转开放；④快速病理提示高度恶性肿瘤或切缘不足，手术采用扩大切除以保证安全切缘；⑤其他不可控因素的存在，手术方案临时改变，可能影响观察指标时。以上标准均以当时患者手术录像进行筛选。

### 1.4 术后处理

术后采用胸外科加速康复（enhanced recovery after surgery, ERAS）标准医疗护理程序，嘱患者清醒状态下采用适宜力度每小时咳嗽6次。手术次日晨行床边胸部正位DR检查，观察肺是否复张良好。观察胸腔闭式引流情况，根据如下标准进行分级：0度无漏气；I度用力咳嗽时漏气；II度深呼吸时漏气；III度平静呼气末即有漏气。

术后拔除胸腔引流管标准：①胸部正位DR片无气胸及包裹性积液；②咳嗽时为0度无漏气状态；③每日胸腔引流量小于300 mL。拔除胸腔引流管后次日行胸部正侧位DR片及血常规检查，检查结果无特殊，患者无不适主诉予以出院。

### 1.5 数据收集

日胸腔引流量、术中漏气分级、胸腔引流管留置时间、术后住院时间、术后血常规、术后常规病理、术后拔除胸管前后的胸片、发生术后肺持续性漏气的比例。

### 1.6 统计学方法

使用GraphPad软件及SPSS 23.0软件进行数据处理及图像合成。所有经筛选纳入研究的患者数据经汇总、分析后，使用*t*检验（Student's *t*-test）及*χ*^2^检验进行统计学分析与比较，*P* < 0.05为差异有统计学意义。

## 2 结果

研究共纳入61例A组患者以及59例B组患者。对所有患者的术前信息进行汇总分析，可见两组患者的年龄、性别、肺功能检查结果及肿瘤标志物6项之间的差异均不具有统计学意义（表 1）。

**1:** 两组患者的术前检查结果 Preoperative examination results of two groups of patients

Items	Group A (*n*=61)	Group B (*n*=59)	*P*
Age (yr)	53.3±9.7	51.9±11.5	0.462
Gender (Male/Female)	18/43	19/40	0.752
Pulmonary function			
VC	3.13±0.56	3.15±0.64	0.810
FEV_1_	98.44±11.92	95.86±15.21	0.300
FEV_1_/FVC	79.65±4.55	80.74±5.48	0.236
MMEF75/25	2.45±0.87	2.63±0.92	0.425
DLCO	6.99±1.16	7.18±1.51	0.441
Tumor marker			
AFP	2.91±1.46	3.15±1.37	0.353
CEA	2.13±0.99	2.43±1.44	0.182
CA19-9	10.88±7.90	11.66±9.31	0.621
CA72-4	4.12±4.17	5.78±7.70	0.143
CYFRA21-1	1.96±0.64	1.89±0.84	0.594
NSE	16.77±4.84	15.53±4.44	0.145
VC: vital capacity; FEV_1_: the first second forced exhalation volume; FEV_1_/FVC: the ratio of forced expiratory volume to forced vital capacity in one second; MMEF75/25: forced mid expiratory velocity; DLCO: carbon monoxide diffusing capacity; AFP: alpha-fetoprotein; CEA: carcinoembryonic antigen; CA19-9: glycoprotein antigen 19-9; CA72-4: glycoprotein antigen 72-4; CYFRA21-1: cytokeratin 19 fragment; NSE: neuron specific enolase.			

两组患者的术中情况（手术时间、术中胸腔引流管放置数目、术中肺漏气等级）相比较其差异亦不具有统计学意义。

术后相关指标的分析统计上，我们发现两组患者的平均胸腔引流量、拔除胸腔引流管前/后的胸片、拔除胸腔引流管后的血常规的差异不具有统计学意义（表 2）；但两组患者的胸腔引流管拔除时间（*P*=0.019）、术后住院时间（*P*=0.017）的差异具有统计学意义（图 1）；而在术后平均每日引流量上，差异不具有统计学意义（*P*=0.145）。

**2:** 两组患者的术后观测指标 Postoperative observation indexes of patients in the two groups

Items	Group A (*n*=61)	Group B (*n*=59)	*P*
Operation time (min)	133.5±25.4	128.4±22.5	0.466
Number of drainage tubes	1	1	
Tumor location			
Left upper lobe	7 (11.5%)	7 (11.9%)	
Left lower lobe	15 (24.6%)	20 (33.9%)	
Right upper lobe	19 (31.1%)	22 (37.3%)	
Right lower lobe	20 (32.8%)	10 (16.3%)	
Intraoperative air leakage grading			0.716
Level 0	28 (45.9%)	27 (45.8%)	
Level 1	29 (47.5%)	30 (50.8%)	
Level 2	4 (6.6%)	2 (3.4%)	
Level 3	0	0	
Pathology			0.811
Benign	2 (3.3%)	4 (6.8%)	
AAH	2 (3.3%)	3 (5.1%)	
AIS	20 (30.8%)	16 (27.1%)	
MIA	8 (13.1%)	10 (16.9%)	
IAC	29 (47.5%)	26 (44.1%)	
Average daily drainage volume (mL)	190.01±56.77	175.70±49.79	0.145
Comparison of chest radiography before/after remove chest tube			0.678
No significant difference	58 (95.1%)	57 (96.6%)	
Minor pneumothorax	3 (4.9%)	2 (3.4%)	
Severe pneumothorax	0	0	
Blood routine after remove chest tube			
WBC (×10^9^/L)	6.92±1.81	6.65±1.56	0.384
NEU (×10^9^/L)	5.62±1.65	5.02±2.44	0.116
NEU (%)	70.71±4.20	69.10±7.33	0.139
PAL (> 72 h)	7 (11.5%)	16 (27.1%)	0.030
Drainage time (d)	2.46±0.70	2.88±1.19	0.019
Postoperative hospital stay (d)	4.08±1.07	4.68±1.58	0.017
AAH: atypical adenomatous hyperplasia; AIS: adenocarcinoma *in situ*; MIA: microinvasive adenocarcinoma; IAC: invasive adenocarcinoma; PAL: prolonged air leak; WBC: white blood cell; NEU: neutrophils.				

**1:**
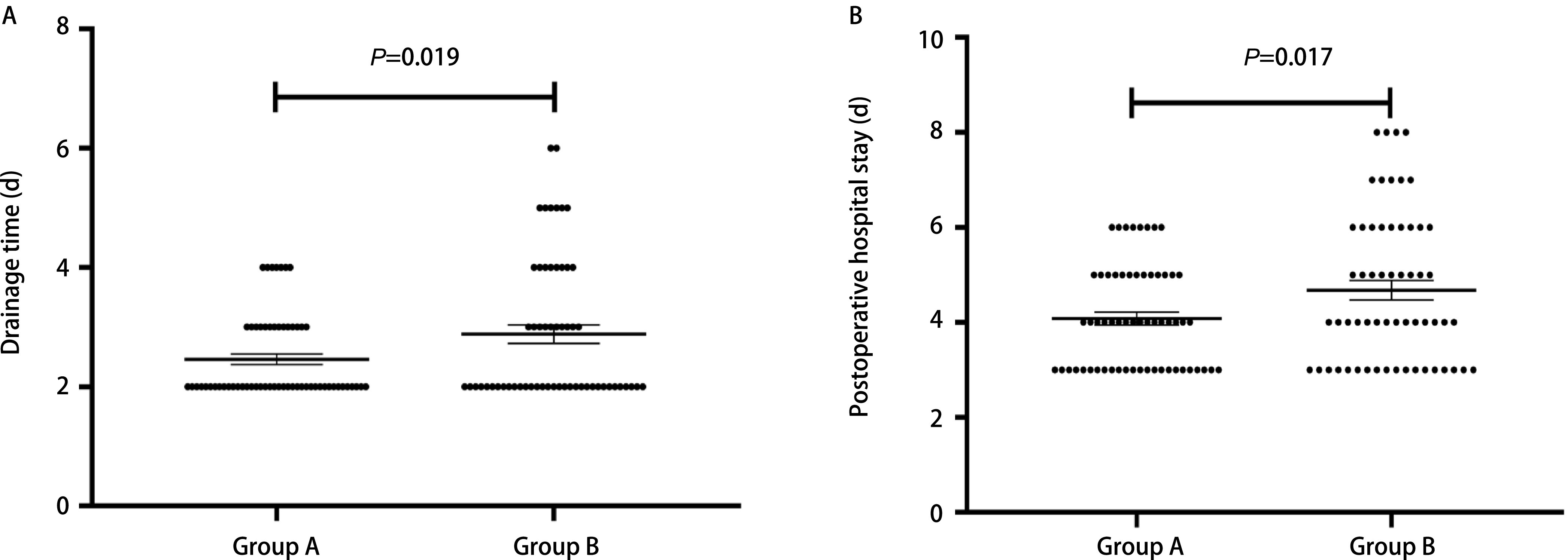
两组患者术后引流及住院时间对比。A：肺段切除术后胸管引流时间；B：肺段切除术后住院时间。 Comparison of postoperative drainage and hospitalization time between the two groups. A: Periods of chest drainage after segmentectomy; B: Periods of hospital stay after segmentectomy.

## 3 讨论

肺术后持续性肺漏气（prolonged air leakage, PAL）是肺部外科手术的主要术后并发症之一^[14]^。术后持续的肺漏气会影响患者术后肺复张及支气管上皮分泌物的排出，将增加术后胸腔感染、胸腔积液的风险，导致术后长期住院^[15]^；持续的漏气会显著延长胸腔引流管置入时间，而长时间的胸腔闭式引流易导致引流管口感染，拔除引流管后切口不愈合等负面影响。

随着电视辅助胸腔镜手术（video-assisted thoracoscopic surgery, VATS）技术的普及，全胸腔镜下解剖性肺段切除术因为保存更多的有效肺容积，对肺功能影响较小等诸多优势^[6]^，在胸外科领域的应用日益广泛，但肺段切除术的术后主要并发症仍然是肺漏气，有研究^[16]^报道称，肺段切除术的平均术后漏气发生率高于肺叶切除术。目前，我科常用的术中修补材料有纤维蛋白粘合胶与PGA补片。虽已有文献^[9]^表明，纤维蛋白粘合胶+三层PGA补片可明显减少术后漏气时间，但由于PGA补片单片较小，价格昂贵且肺段切除术段间交界面的创面较大，所以PGA补片的应用受到较大限制，因此，目前临床操作中的常用修补方式为纤维蛋白粘合胶+单层PGA补片。

微孔多糖止血粉作为一种新的植物性止血材料，常用于术中毛细血管渗血的创面止血，效果较为肯定^[10]^。由于解剖性肺段切除术需先分离靶段动静脉及支气管，且段间交界面较大，故于临床实际操作中难免会造成分离平面附近的肺组织或终末细支气管、肺小动静脉、肺泡毛细血管的损伤，从而导致创面的出血及漏气。因此，我中心在行解剖性肺段切除术时，对于段间交界面的处理常为先予以微孔多聚糖止血粉均匀喷洒创面，后使用纤维蛋白粘合胶+单层PGA补片进行修补。

由回顾性分析的结果看，A组的患者相比于B组，具有更短的术后引流时间。胸腔引流管的拔除指证主要在于肺复张、不漏气及渗出少三个要素。而两组患者的术后平均每日引流量无明显差异则说明了使用微孔多聚糖止血粉并不会显著影响术后渗出，同样两组患者的术后胸片的定性对比也证明了两组患者发生术后肺不张的差异不具有统计学意义，且两组患者术后测漏的漏气分级对比无统计学意义，可认为两组患者运用修补材料之前的漏气程度一致，因此两组患者的胸腔引流管拔除时间长短主要源于漏气的差异性。而两组患者拔除胸腔引流管后，血常规中的白细胞计数、中性粒细胞计数/比例的差异不具有统计学意义，从而排除了因术后感染或炎症反应干扰术后住院时间的可能；两组患者在拔除胸腔引流管后，均未见显著气胸发生的案例，可认为两组患者的术后住院时间差异这一指标可以间接反映术后漏气的发生情况。综上，可认为肺段切除术中创面的修补采用微孔多聚糖止血粉+纤维蛋白粘合胶+单层PGA补片的方式对于减少术后肺漏气的效果要比使用纤维蛋白粘合胶+单层PGA补片的更加有效。

尽管本研究发现术中采用微孔多聚糖止血粉+纤维蛋白粘合胶+单层PGA补片的段间平面修补方式对于减少术后肺漏气的效果可观，但是这项研究亦具有其局限性。首先，样本量不足。在本研究中A组61例，B组59例，样本不足且患者主观因素较多，可能会对本研究结果造成一定的影响，如不当饮食影响术后引流；其次，此研究为回顾性分析单个肺段切除术段间平面修补材料的对比研究，对于多发结节等需要二次手术的患者，是否会影响其再次手术的效果尚未可知，故对于此类患者其术中修补材料的最佳方案亦有待临床大规模的随机对照研究来进一步验证；最后，微孔多聚糖止血粉作为止血材料，其对于肺段切除术中段间交界面的止漏原理尚未可知，其效果需要更多的理论支撑。

综上，微孔多聚糖止血粉在肺段切除术中既可以用于对创面快速止血，又可以作为肺组织创面的修补材料。但是，仍需要探究微孔多聚糖止血粉对于肺间平面的修补止漏原理，对于多发肺结节等需要二次手术的患者，其术中修补材料的选择亦需要根据其具体情况来判断，并非适应于全部患者。此外减少术后肺漏气发生需要的是综合的治疗措施，也期待更多对于减少肺漏气的研究探索，以减少患者术后肺漏气的发生及住院时间。
